# The Synergism of PGN, LTA and LPS in Inducing Transcriptome Changes, Inflammatory Responses and a Decrease in Lactation as Well as the Associated Epigenetic Mechanisms in Bovine Mammary Epithelial Cells

**DOI:** 10.3390/toxins12060387

**Published:** 2020-06-11

**Authors:** Yongjiang Wu, Yawang Sun, Xianwen Dong, Jingbo Chen, Zili Wang, Juncai Chen, Guozhong Dong

**Affiliations:** 1 College of Animal Science and Technology, Southwest University, Beibei District, Chongqing 400716, China; wuyongjiang@email.swu.edu.cn (Y.W.); syaw507@swu.edu.cn (Y.S.); chenjingbooo@outlook.com (J.C.); wzl9698@swu.edu.cn (Z.W.); juncaichen@swu.edu.cn (J.C.); 2 Institute for Herbivorous Livestock Research, Chongqing Academy of Animal Science, Chongqing 402460, China; chenghuafen@email.swu.edu.cn

**Keywords:** lipopolysaccharide, peptidoglycan, lipoteichoic acid, DNA methylation, histone acetylation, inflammation, lactation, bovine mammary epithelial cells

## Abstract

Mastitis is usually caused by a variety of pathogenic bacteria that include both Gram-positive and Gram-negative bacteria. Lipopolysaccharide (LPS) is the pathogen-associated molecular pattern (PAMP) of Gram-negative bacteria, and peptidoglycan (PGN) and lipoteichoic acid (LTA) are those of Gram-positive bacteria. The effects of LPS, PGN and/or LTA on inflammatory response and lactation in bovine mammary epithelial cells (BMECs) are well studied, but the epigenetic mechanisms of their effects received less attention. Furthermore, since the three PAMPs are often simultaneously present in the udder of cows with mastitis, it has implications in practice to study their additive effects. The results show that co-stimulation of bovine mammary epithelial cells with PGN, LTA, and LPS induced a higher number of differentially expressed genes (DEGs) and greater expressions of inflammatory factors including interleukin (IL)-1β, IL-6, IL-8, tumor necrosis factor-α (TNF-α), chemokine (C-X-C motif) ligand (CXCL)1, and CXCL6. In addition, co-stimulation further increased DNA hypomethylation compared with sole LPS stimulation. Co-stimulation greatly decreased casein expression but did not further decrease histone acetylation levels and affect the activity of histone acetyltransferase (HAT) and histone deacetylase (HDAC), compared with sole LPS stimulation. Collectively, this study demonstrated that PGN, LTA, and LPS had an additive effect on inducing transcriptome changes and inflammatory responses in BMECs, probably through inducing a greater decrease in DNA methylation. Co-stimulation with PGN, LTA, and LPS decreased casein expression to a greater degree, but it might not be linked to histone acetylation and HAT and HDAC activity.

## 1. Introduction 

Mastitis is one of the most common and prevalent inflammatory diseases in dairy cows. It continues to be the number one cause of economic losses to the dairy industry, due to a decrease in milk yield and quality as well as an increase in the cost of treatment and the cull rate of cows [1,2]. For instance, the economic cost associated with mastitis approached 2 billion dollars and exceeded 1.55 billion euros per year in the USA and Europe, respectively [3,4]. 

In dairy cows, mastitis is usually caused by multiple pathogenic bacteria that invade the udder, multiply in the milk-producing tissues, and produce toxins that are the immediate cause of udder injury [5]. These pathogenic bacteria include both Gram-positive bacteria, e.g., *Staphylococcus aureus* (*S. aureus*), *Streptococcus agalactiae*, *Streptococcus* spp., etc., as well as Gram-negative bacteria, such as *Escherichia coli* (*E. coli*), *Klebsiella* spp., *Enterobacter* spp., etc. [6,7,8,9,10]. *E. coli* and *S. aureus* are amongst the most common and important pathogens to induce mastitis [1,11]. Lipopolysaccharide (LPS) is the pathogen-associated molecular pattern (PAMP) of Gram-negative bacteria, and lipoteichoic acid (LTA) and peptidoglycan (PGN) are those of Gram-positive bacteria [2]. Previous studies have reported the effects of LPS, PGN, LTA, and PGN + LTA on the gene expression profiles of bovine mammary epithelial cells (BMECs) and mainly focused on their proinflammatory activity [12,13,14,15,16,17,18,19]. However, the additive effects of LPS, PGN, and LTA on the gene expression profiles of BMECs are still unclear. Since these three PAMPs are commonly present at the same time in the udders of cows, especially under the condition of mastitis. Therefore, this has implications in the practice of studying their synergism in inducing inflammation, altering gene expression profiles, and decreasing lactation in BMECs. In addition, the epigenetic mechanisms of their effects on inflammation and lactation of BMECs need to be investigated.

The epigenetic modification mechanisms mainly include DNA methylation, histone tail modification, and non-coding RNA regulation, all of which modulate gene expression without changing the DNA sequence [20,21,22]. DNA hypermethylation is generally associated with gene silencing [23,24]. DNA methylation is catalyzed by DNA methyltransferase (DNMT) in mammals [25,26]. On the other hand, higher acetylation levels of histones are associated with the activation of transcription [27]. Histone acetylation is catalyzed by histone acetyltransferase (HAT), whereas histone deacetylation is catalyzed by histone deacetylase (HDAC) [28,29]. The gene expression of inflammatory factors could be regulated by epigenetic modifications [30,31]. LPS could cause DNA hypomethylation at many inflammatory loci by suppressing DNMT expression, and then increase the inflammatory factor expression of human dental pulp cells [32] and macrophages [33], rat brain tissue [34], bovine fibroblasts [35], and so on. Our previous study showed that LPS, LTA, and PGN enhanced the inflammatory responses of BMECs by decreasing DNA methylation levels [19,36]. Other studies have also shown that LTA and PGN could cause the DNA hypomethylation of the key regulators of inflammatory pathways, promoting the release of a variety of inflammatory factors [37,38]. In addition, our previous studies showed that LPS, LTA, and PGN suppressed the expression of lactation-related genes of BMECs due to reducing histone H3 acetylation through regulating HAT and HDAC activity [19,39]. Thus, we speculated that co-stimulation with LPS, LTA, and PGN might have an additive effect on DNA hypomethylation and histone hypoacetylation, producing a more intense inflammatory response and decreasing casein expression to a greater degree than single stimulation of either of the PAMPs in BMECs. 

Therefore, this present study aims to investigate the additive effects of PGN, LTA and LPS on the gene expression profile associated with inflammation and lactation in the BMECs. Meanwhile, the inherent epigenetic mechanisms were also explored. 

## 2. Results

### 2.1. Effects of LPS Stimulation and Co-Stimulation with PGN, LTA and LPS (PLL) on Transcriptome 

#### 2.1.1. Overview of RNA Sequencing (RNA-Seq) Data 

**Figure 1:**
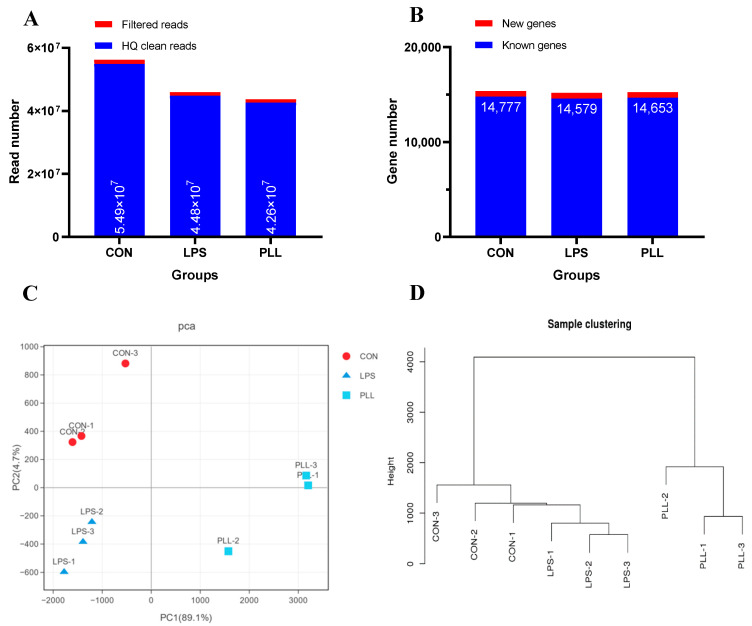
The statistics of gene expression profiles. (**A**) The number of filtered and high-quality (HQ) clean reads. (**B**) The number of new and known genes. (**C**) The principle component analysis (PCA) plot of global gene expression profiles of the samples. (**D**) The sample clustering tree. CON, control group; LPS, lipopolysaccharide group; PLL, the group of co-stimulation with peptidoglycan (PGN), lipoteichoic acid (LTA), and LPS.

After RNA-Seq, a total of 4–6 million raw sequencing reads were generated in each group. The high-quality (HQ) clean reads obtained accounted for more than 99% of all the raw reads (Figure 1A) and were mapped to the bovine reference genome (*Bos Taurus*, assembly ARS-UCD1.2). The mean mapping ratio was more than 93% in each group. A total of 14,777, 14,597, and 14,653 known genes and 585, 585, and 587 new genes were identified in the control (CON), LPS, and PLL groups, respectively (Figure 1B). The cumulative variance contribution rate (94.1%, PC1 + PC2) of the principle component analysis (PCA) for the gene expression profiles was higher than the standard of 85% (Figure 1C). The three replicates in each group were closely clustered, and among the three groups, they were well separated (Figure 1C). The sample clustering analyses further confirmed the PCA results (Figure 1D).

**Figure 2:**
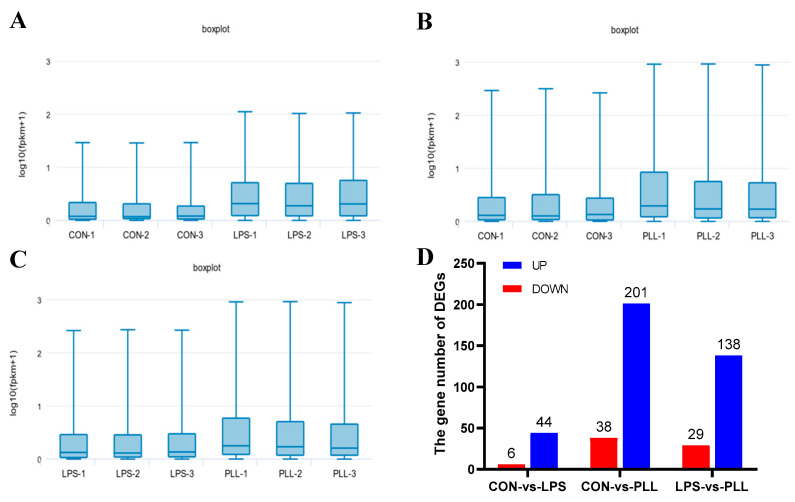
The statistics of gene expression levels and differentially expressed genes (DEGs). (**A**) The boxplots of gene expression levels in the CON and LPS groups. (**B**) The boxplots of gene expression levels in the CON and PLL groups. (**C**) The boxplots of gene expression levels in the LPS and PLL groups. (**D**) The statistics of the number of DEGs in the three comparative conditions (CON vs. LPS, CON vs. PLL, and LPS vs. PLL). CON, control group; LPS, lipopolysaccharide group; PLL, the group of co-stimulation with peptidoglycan (PGN), lipoteichoic acid (LTA), and LPS; log_10_ (fpkm + 1), log_10_ (fragments per kilobase million + 1); UP, up-regulated DEGs; DOWN, down-regulated DEGs.

The differential analysis of the gene expression levels between groups was carried out using the Edge R software. Genes with a false-discovery rate (FDR) of <0.05 and an absolute value of log_2_ fold change (|log_2_ FC|) > 1 were considered as differentially expressed genes (DEGs). The boxplots of gene expression levels (log_10_ (fragments per kilobase million + 1)) for each sample are shown in Figure 2A–C. The gene expression levels of the PLL group were higher than the LPS group, and they were both higher than the CON group (Figure 2A–C). The gene number for either the up- or down-regulated DEGs in the CON vs. PLL condition was more than those in the CON vs. LPS and LPS vs. PLL conditions (Figure 2D).

#### 2.1.2. Gene Ontology (GO) Function Annotation and Kyoto Encyclopedia of Genes and Genomes (KEGG) Pathway Enrichment Analyses of DEGs

**Figure 3:**
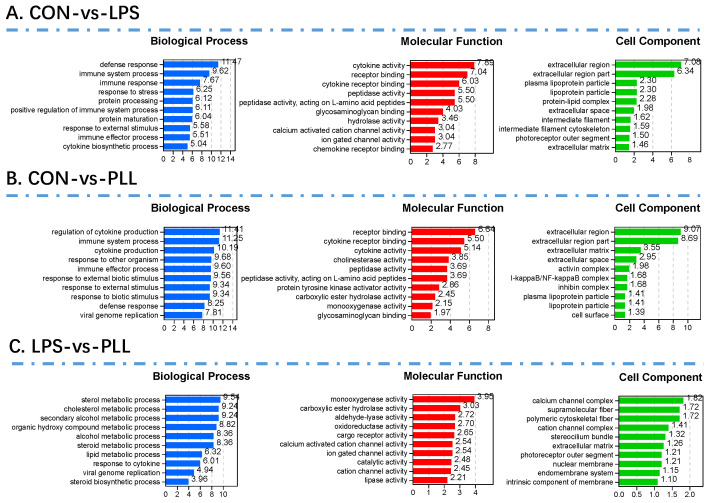
The top 10 Gene Ontology (GO) terms enriched by the differentially expressed genes (DEGs) in the three comparative conditions (CON vs. LPS, CON vs. PLL, and LPS vs. PLL). (**A**) The histograms of the top 10 GO terms enriched by the DEGs in the CON vs. LPS condition. (**B**) The histograms of the top 10 GO terms enriched by the DEGs in the CON vs. PLL condition. (**C**) The histograms of the top 10 GO terms enriched by the DEGs in the LPS vs. PLL condition. CON, control group; LPS, lipopolysaccharide group; PLL, the group of co-stimulation with peptidoglycan (PGN), lipoteichoic acid (LTA), and LPS.

For further insight into the biological function and distribution of these DEGs, GO function annotation and enrichment analyses (*p* < 0.05) were performed. The GO categories included biological processes (BP), cell components (CC), and molecular functions (MF), represented with different colors (Figure 3). Only the top 10 GO terms are presented in Figure 3. The DEGs in the CON vs. LPS condition seem to mainly fall into the extracellular region, function in cytokine activity and cytokine receptor binding, and participate in defense responses and immune system processes (Figure 3A). The GO enrichment results of the DEGs in the CON vs. PLL condition (Figure 3B) were basically consistent with those of the CON vs. LPS condition, indicating the similar biological functions of LPS and PLL. The DEGs in the LPS vs. PLL condition might mainly fall into the calcium channel complex, function in monooxygenase activity, and participate in the sterol metabolic process (Figure 3C).

**Figure 4:**
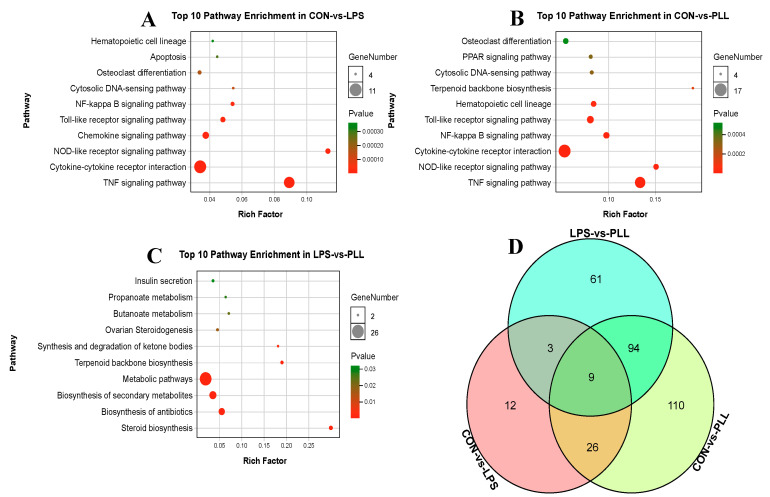
The top 10 pathways enriched by the differentially expressed genes (DEGs) and the Venn diagram of the DEGs in the three comparative conditions (CON vs. LPS, CON vs. PLL, and LPS vs. PLL). (**A**) The top 10 pathways enriched by the DEGs in the CON vs. LPS condition. (**B**) The top 10 pathways enriched by the DEGs in the CON vs. PLL condition. (**C**) The top 10 pathways enriched by the DEGs in the LPS vs. PLL condition. (**D**) The Venn diagram of the DEGs among the three conditions. CON, control group; LPS, lipopolysaccharide group; PLL, the group of co-stimulation with peptidoglycan (PGN), lipoteichoic acid (LTA), and LPS.

Meanwhile, all the DEGs in the three conditions (CON vs. LPS, CON vs. PLL, and LPS vs. PLL) were mapped to the KEGG database to detect the significant enrichment of pathways altered by LPS and PLL. Only the top 10 most significant pathways were presented by the bubble charts in Figure 4. The DEGs in the CON vs. LPS condition were mainly significantly enriched in the tumor necrosis factor (TNF) signaling pathway, the cytokine–cytokine receptor interaction, the nucleotide-binding oligomerization domain (NOD)-like receptor signaling pathway, the chemokine signaling pathway, the Toll-like receptor (TLR) signaling pathway, and the nuclear factor-kappa B (NF-κB) signaling pathway (Figure 4A). The significantly enriched pathways of the DEGs in the CON vs. PLL condition were basically consistent with those in the CON vs. LPS condition, but the gene number of the DEGs enriched in those pathways in the CON vs. PLL condition was more than in the CON vs. LPS condition (Figure 4B). The DEGs in the LPS vs. PLL condition were mainly significantly enriched in metabolic pathways and biosynthesis of secondary metabolites (Figure 4C). The DEG overlap among the three conditions (CON vs. LPS, CON vs. PLL and LPS vs. PLL) is presented using a Venn diagram (Figure 4D). Between the CON vs. LPS and CON vs. PLL condition, there were 35 common DEGs (Table S1). Among the three conditions (CON vs. LPS, CON vs. PLL and LPS vs. PLL), there were nine common DEGs (Table S2). There were 12 DEGs that only appeared in the condition of CON vs. LPS (Table S3).

As shown in Table 1, both LPS and PLL promoted the expression of immune-related DEGs. The gene number and the fold change in the expression of the immune-related DEGs induced by the PLL were greater than those by the LPS, indicating that co-stimulation with PGN, LTA, and LPS induced more intense immune or inflammatory responses than the sole LPS stimulation.

### 2.2. Validation of RNA-Seq Data by Reverse Transcription Quantitative Real-Time Polymerase Chain Reaction (RT-qPCR) Analyses

**Figure 5:**
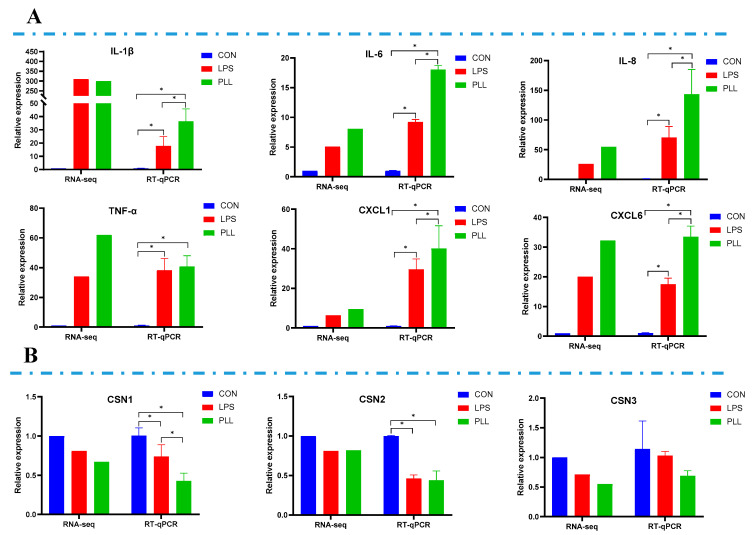
Validation of RNA sequencing (RNA-Seq) data by reverse transcription quantitative real-time polymerase chain reaction (RT-qPCR) analyses. (**A**) The relative mRNA expression of six inflammation-related genes measured by RNA-Seq and RT-qPCR. (**B**) The relative mRNA expression of three casein genes measured by RNA-Seq and RT-qPCR. Cells were collected 24 h after the stimulation to isolate RNA for RNA-Seq and RT-qPCR. The data are presented as mean ± standard deviation (SD). The error bars represent standard deviation, and the asterisk indicates statistical difference (*n* = 6, * *p* < 0.05) between the indicated columns, based on one-way analysis of variance followed by Duncan’s multiple comparison test. *IL-1β*, interleukin-1β; *IL-6*, interleukin-6; *IL-8*, interleukin-8; *TNF-α*, tumor necrosis factor-α; *CXCL1*, chemokine (C-X-C motif) ligand 1; *CXCL6*, chemokine (C-X-C motif) ligand 6; *CSN1S1*, αS1-casein; *CSN2*, β-casein; *CSN3*, κ-casein; CON, control group; LPS, lipopolysaccharide group; PLL, the group of co-stimulation with peptidoglycan (PGN), lipoteichoic acid (LTA), and LPS.

In order to validate the RNA-Seq data, the relative mRNA expression of six inflammation-related genes including interleukin (*IL*)*-1β*, *IL-6*, *IL-8*, tumor necrosis factor-α (*TNF-α*), chemokine (C-X-C motif) ligand (*CXCL*)*1* and *CXCL6*, as well as three casein genes, namely αS1-casein (*CSN1S1*), β-casein (*CSN2*) and κ-casein (*CSN3*), was measured by RT-qPCR. As shown in Figure 5, the RT-qPCR results are basically consistent with the RNA-Seq data. The relative mRNA expression of inflammation-related genes of the three groups increased in the following order: CON < LPS < PLL (Figure 5A). Compared with the CON group, the relative mRNA expression of *CSN1S1* and *CSN2* in the LPS and PLL groups significantly decreased (Figure 5B). The relative mRNA expression of *CSN3* in the LPS and PLL groups was also numerically lower than in the CON group, although these differences were not significant (Figure 5B).

### 2.3. LPS and PLL Reduced Global DNA Methylation Levels by Suppressing DNMT Activity

**Figure 6:**
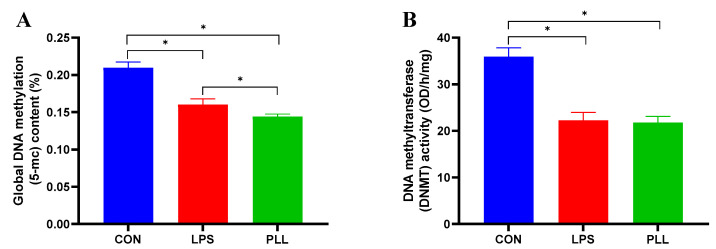
The methylation levels of DNA and enzyme activity of methyltransferase (DNMT) measured by enzyme-linked immunosorbent assay (ELISA). (**A**) The methylation levels of DNA. (**B**) The enzyme activity of DNMT. Cells were collected 24 h after the stimulation to isolate DNA and nucleoprotein for ELISA. The data are presented as mean ± standard deviation (SD). The error bars represent standard deviation, and the asterisk indicates statistical difference (*n* = 6, * *p* < 0.05) between the indicated columns, based on one-way analysis of variance followed by Duncan’s multiple comparison test. CON, control group; LPS, lipopolysaccharide group; PLL, the group of co-stimulation with peptidoglycan (PGN), lipoteichoic acid (LTA), and LPS.

The global DNA methylation levels and DNMT activity of cell samples in each group are presented in Figure 6. The global DNA methylation levels and DNMT activity of cell samples in the LPS and PLL groups significantly decreased, compared with the CON group. In addition, the global DNA methylation levels of cell samples in the PLL group were significantly lower than those in the LPS group. 

### 2.4. LPS and PLL Reduced Histone H3 Acetylation Levels by Suppressing HAT Activity and Promoting HDAC Activity

**Figure 7:**
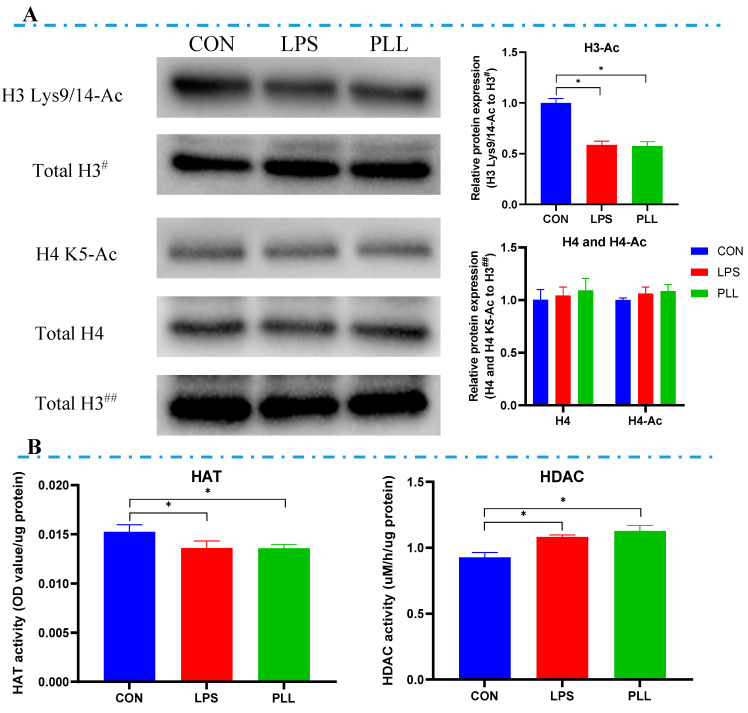
The acetylation levels of histones (H3 and H4) and the activity of histone acetyltransferase (HAT) and histone deacetylase (HDAC). (**A**) Histone acetylation (H3 Lys9/14-Ac and H4 K5-Ac) levels detected by Western blot and quantification analysis. Protein loading was normalized to histone H3 protein which served as an internal control. Total H3^#^ is an internal control for H3 Lys9/14-Ac. Total H3^##^ is an internal control for histone H4 and H4 K5-Ac. Quantitation of blots was performed by densitometric analysis and was representative of three independent trials. (**B**) HAT and HDAC activity measured by enzyme-linked immunosorbent assay (ELISA). Cells were collected 24 h after the stimulation to isolate nucleoprotein for Western blot and ELISA (*n* = 6). The data are presented as mean ± standard deviation (SD). The error bars represent standard deviation, and the asterisk indicates statistical difference (* *p* < 0.05) between the indicated columns, based on one-way analysis of variance followed by Duncan’s multiple comparison test. CON, control group; LPS, lipopolysaccharide group; PLL, the group of co-stimulation with peptidoglycan (PGN), lipoteichoic acid (LTA), and LPS.

The acetylated histone H3 (H3 Lys9/14-Ac) content in the LPS and PLL groups were significantly lower than in the CON group, while the differences in acetylated histone H4 (H4 K5-Ac) content among all the groups were not significant (Figure 7A). Compared with the CON group, significantly decreased HAT activity and significantly increased HDAC activity were observed in the LPS and PLL groups (Figure 7B).

### 2.5. LPS and PLL Promoted Inflammation but Reduced Lactation 

#### 2.5.1. LPS and PLL Increased Inflammatory Factor Release 

**Figure 8:**
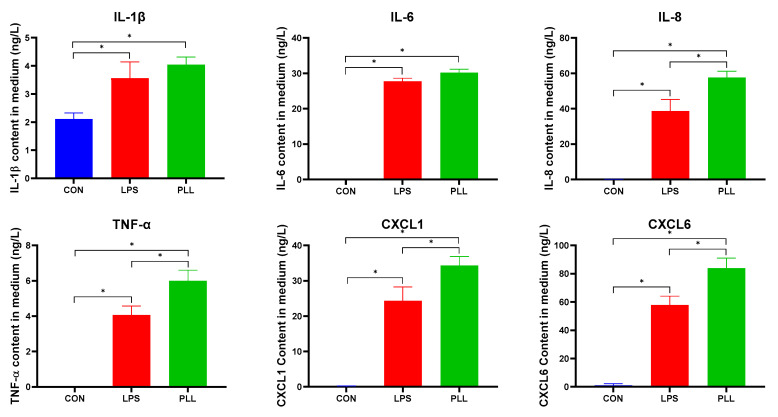
The inflammatory factor concentrations measured by enzyme-linked immunosorbent assay (ELISA). Medium supernatant was collected 24 h after the stimulation for ELISA assays. The data are presented as mean ± standard deviation (SD). The error bars represent standard deviation, and the asterisk indicates statistical difference (*n* = 6, * *p* < 0.05) between the indicated columns, based on one-way analysis of variance followed by Duncan’s multiple comparison test. IL-1β, interleukin-1β; IL-6, interleukin-6; IL-8, interleukin-8; TNF-α, tumor necrosis factor-α; CXCL1, chemokine (C-X-C motif) ligand 1; CXCL6, chemokine (C-X-C motif) ligand 6; CON, control group; LPS, lipopolysaccharide group; PLL, the group of co-stimulation with peptidoglycan (PGN), lipoteichoic acid (LTA), and LPS.

As shown in Figure 8, the inflammatory factors were nearly not released in the CON group. All the inflammatory factor concentrations in the LPS and PLL groups significantly increased, compared with the CON group. All the inflammatory factor concentrations in the PLL group were higher than in the LPS group. The pattern of the inflammatory factor release was basically consistent with the results obtained in the RT-qPCR and RNA-seq analyses above. 

#### 2.5.2. LPS and PLL Decreased Casein Synthesis

**Figure 9:**
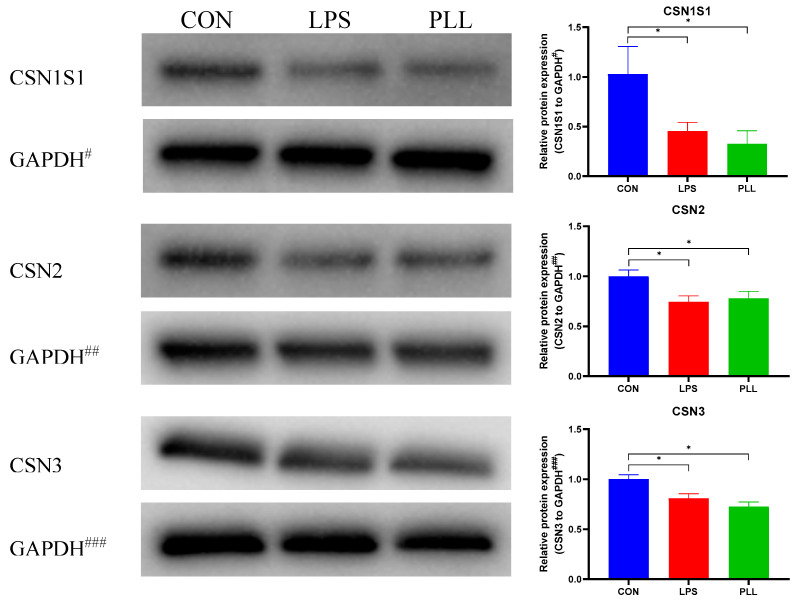
The three casein (CSN1S1, CSN2, and CSN3) protein expression detected by Western blot and quantification of protein expression. Protein loading was normalized to glyceraldehyde-3-phosphate dehydrogenase (GAPDH) protein which served as an internal control. GAPDH^#^, GAPDH^##^ and GAPDH^###^ are the internal controls for CSN1S1, CSN2 and CSN3, respectively. Quantitation of blots was performed by densitometric analysis and was representative of three independent trials. Cells were collected 24 h after the stimulation to isolate total protein for Western blot. The data are presented as mean ± standard deviation (SD). The error bars represent standard deviation, and the asterisk indicates statistical difference (* *p* < 0.05) between the indicated columns, based on one-way analysis of variance followed by Duncan’s multiple comparison test. CSN1S1, αS1-casein; CSN2, β-casein; CSN3, κ-casein; CON, control group; LPS, lipopolysaccharide group; PLL, the group of co-stimulation with peptidoglycan (PGN), lipoteichoic acid (LTA), and LPS.

As shown in Figure 9, the protein expression of three major caseins (CSN1S1, CSN2, and CSN3) was detected by Western blot to analyze the effects of LPS and PLL on milk protein synthesis in BMECs. The protein expression of all the three caseins in the LPS and PLL groups significantly decreased, compared with the CON group. The protein expression of the CSN1S1 and CSN2 in the PLL group was lower than in the LPS group, although the differences were not significant.

## 3. Discussion 

The occurrence and development of mastitis are usually caused by varied and multifaceted bacterial pathogens invading the mammary gland [6,7,8,9]. *E. coli* and *S. aureus* are the two most common bacterial pathogens causing mastitis [1,9,11]. Their cell wall components, such as LPS, LTA, and PGN, released during the process of proliferation or/and after death, can separately induce inflammatory responses and reduce lactation in the mammary gland [40,41,42,43,44,45,46]. In practical production, the mammary glands of cows suffering mastitis are usually infected with more than one kind of pathogenic bacteria [1,9]. Thus, LPS, LTA, and PGN are commonly present simultaneously in the udders, thereby aggravating inflammation. Therefore, it is very important to study the addictive effects of LPS, LTA, and PGN on inflammation and lactation and to reveal the relevant mechanisms in BMECs.

In the present study, the transcriptome changes after co-stimulation with PGN, LTA, and LPS were detected using the RNA-Seq technology combined with bioinformatics analyses. The effects of *E. coli*-derived LPS on the transcriptome of BMECs in vivo and in vitro have been reported in previous studies. In one study, the cow mammary tissue was challenged for 2.5 h with 10 μg/mL LPS in vivo, which resulted in 20 down-regulated and 169 up-regulated DEGs, and significantly activated NOD-like receptor signaling, TLR signaling, retinoic inducible gene (RIG)-I-like receptor signaling, and apoptosis pathways [15]. In another study, 20 μg/mL LPS stimulation of BMECs for 3 h and 6 h in vitro caused 16 down-regulated and 201 up-regulated DEGs, and 273 down-regulated and 541 up-regulated DEGs, respectively [16]. The DEGs were significantly enriched in the transcription and activation pathways and cytokine and chemokine pathways [16]. In addition, 10 μg/mL LPS treatment of BMECs for 12 h in vitro caused 483 down-regulated and 536 up-regulated DEGs [12]. The DEGs were mainly enriched in inflammation relative pathways, such as the TLR, the NF-κB, and the NOD-like receptor signaling pathways [12]. In our study, the BMECs were stimulated with 0.1 μg/mL LPS for 24 h in vitro, inducing 6 down-regulated and 44 up-regulated DEGs. The DEGs were mainly enriched in the inflammation-related pathways, such as the TNF signaling pathway, the cytokine–cytokine receptor interaction, the NOD-like receptor signaling pathway, the chemokine signaling pathway, the TLR signaling pathway, and the NF-κB signaling pathway. Therefore, although the number of DEGs after LPS treatment was different in different studies due to different doses and treatment time, all the main pathways enriched by DEGs were associated with inflammation. 

The transcriptome alterations of the BMECs stimulated with LPS or LTA had also been investigated in a previous study [13]. LPS caused 5 down-regulated and 95 up-regulated DEGs, whereas LTA caused 12 down-regulated and 12 up-regulated DEGs [13]. Although the number and the expression of DEGs induced by LPS and LTA were significantly different, the majority of DEGs were enriched in the cytokine–cytokine receptor interaction, the NF-κB signaling pathway, and the NOD-like receptor signaling pathway [13]. After co-stimulation of BMECs for 1 h with PGN (30 μg/mL) and LTA (30 μg/mL), 14 inflammatory mediator-related DEGs and 17 inflammation-related DEGs were found, and the NF-κB signaling pathway was activated [18]. Our previous study indicated that co-stimulation with PGN (30 μg/mL) and LTA (30 μg/mL) induced more intense transcriptome changes than single stimulation in BMECs, and they displayed an additive effect on proinflammatory activity [19]. In the present study, we further analyzed the effects of co-stimulation with PGN (30 μg/mL), LTA (30 μg/mL), and LPS (0.1 μg/mL) on the transcriptome of BMECs by RNA-Seq and found 38 down-regulated and 201 up-regulated DEGs. Although the pathways significantly enriched by the DEGs induced by co-stimulation were basically consistent with those of DEGs induced by sole LPS stimulation, the number of the DEGs induced by co-stimulation was more than by sole LPS stimulation in these pathways. Furthermore, the fold change of immune- or inflammation-related DEGs induced by co-stimulation was also greater than by sole LPS stimulation. Moreover, through RT-qPCR and ELISA analyses, it was found that the mRNA and protein expressions of inflammation-related genes induced by co-stimulation were significantly higher than by sole LPS stimulation. The results indicate the synergism of PGN, LTA, and LPS in inducing a greater magnitude of transcriptome changes and inflammatory responses, and the proinflammatory activity of co-stimulation with PGN, LTA, and LPS also displayed an additive effect.

The gene expression of inflammatory factors usually was regulated by DNA methylation [47,48,49]. Bacterial pathogens have an ability to directly influence the DNA methylation status of a host and then cause the development of inflammatory diseases. For example, the blood neutrophils of cows with mastitis infected by *E. coli* displayed lower DNA methylation levels compared with healthy cows [50]. The DNA methylation levels of inflammation-related genes of peripheral blood lymphocytes in cows with *S. aureus* subclinical mastitis were lower than in healthy cows [51]. In addition, LPS, PGN and LTA, as the cell wall components of bacterial pathogens, could also affect the host DNA methylation. Our previous study demonstrated that LPS at a dose of 0.1 μg/mL caused hypomethylation of immune-related genes in BMECs, through the analysis of genome-wide DNA methylation [36]. LTA induced myeloid differentiation factor 88 (*MyD88*) hypomethylation by decreasing DNMT-1 expression, which resulted in the up-regulation of *MyD88*, activated NF-B pathway, and the subsequent release of LTA-induced inflammatory cytokines in human odontoblast-like cells [37]. PGN could induce more severe inflammatory responses, when promoter methylation of TLR-2 gene was decreased in cystic fibrosis bronchial epithelial cells [38]. In our previous study [19], LTA (30 μg/mL) and PGN (30 μg/mL) + LTA (30 μg/mL) induced DNA hypomethylation, and then caused transcriptome changes and up-regulation of inflammatory factors. In this study, we further analyzed the effects of co-stimulation with PGN (30 μg/mL), LTA (30 μg/mL) and LPS (0.1 μg/mL) on DNA methylation of BMECs and found that both sole LPS stimulation and co-stimulation with PGN, LTA, and LPS decreased DNA methylation levels by suppressing DNMT activity. The co-stimulation reduced DNA methylation more than sole LPS stimulation and induced a more intense inflammatory response in BMECs. Hence, the results corroborate the findings of previous studies and further show that co-stimulation with PGN, LTA, and LPS had an additive effect on DNA hypomethylation and inflammatory responses in BMECs. 

In contrast to DNA hypermethylation, histone hyperacetylation activates gene transcription [52,53]. Histones H3 and H4 are the two most common histones, and their acetylation levels could be regulated by bacterial pathogens. For example, uropathogenic *E. coli* could epigenetically silence the expression of BIM (a BH3 only member of the BCL2 family) by decreasing histone H4 acetylation at the BIM promoter site [54]. Persistent peripheral presence of *S. aureus* reduced histone H3 acetylation in rat brain tissues [55]. In addition to the direct effects of bacterial pathogens on histone acetylation, we found in our previous study that LPS (0.1 μg/mL) could suppress lactation-related gene expression through reducing histone H3 acetylation by enhancing HDAC activity in BMECs [39]. In this study, LPS (0.1 μg/mL) significantly decreased histone H3 acetylation and the expression of three caseins, which was consistent with our previous research. In addition to LPS, histone acetylation was also affected by PGN and LTA. PGN could have an impact on the recruitment of drosophila histone deacetylase 1 (dHDAC1), and LTA could bind closely to arginine-rich histone H3 and H4 [56,57]. Additionally, in our previous study, PGN (30 μg/mL) and LTA (30 μg/mL), alone and combined, suppressed the gene and protein expression of caseins due to decreasing histone H3 acetylation levels through inhibiting HAT or/and increasing HDAC activity in BMECs [19]. Therefore, we speculated that co-stimulation with PGN, LTA, and LPS might further decrease histone acetylation and casein expression of BMECs. In this study, co-stimulation with PGN, LTA, and LPS decreased the gene and protein expression of CSN1S1 and CSN3 of BMECs more than the sole LPS stimulation, which partially supports the above speculation. However, co-stimulation did not further decrease histone acetylation levels and affect HAT and HDAC activity, compared with sole LPS stimulation, indicating that the co-stimulation did not display an additive effect on histone acetylation.

## 4. Conclusions

In summary, co-stimulation with PGN, LTA, and LPS synergistically induced transcriptome changes and inflammatory responses in BMECs, probably through causing a greater decrease in DNA methylation. The co-stimulation also had a synergism in decreasing casein expression to a greater degree, but this might not be associated with histone acetylation as well as HAT and HDAC activity.

## 5. Materials and Methods

### 5.1. Cell Culture and Treatments

Cells of the bovine mammary epithelial cell line MAC-T were seeded onto 6-well plates (about 1 × 10^5^ cells/well), and then incubated in complete culture medium under standard conditions (37 °C, 5% CO_2_) in an incubator. The detailed description of the source and culture method of the cells were given in our previous study [19] and that of Huynh et al. [58]. When MAC-T cells grew to approximately 70% confluence, they were treated for 24 h with no LPS (CON), 0.1 μg/mL LPS (*E. coli* O111:B4, Sigma-Aldrich, L2630, St. Louis, MO, USA), and 30 μg/mL PGN (*S. aureus*, Sigma-Aldrich, 77140, St. Louis, MO, USA) + 30 μg/mL LTA (*S. aureus*, Sigma-Aldrich, L2515, St. Louis, MO, USA) + 0.1 μg/mL LPS (PLL) in the culture medium, respectively. Previous studies of ours and others have shown that 30 μg/mL PGN or LTA and 0.1 μg/mL LPS can effectively induce the immune responses in BMECs in vitro [18,19,36,39]. There were six replicates per treatment or group (*n* = 6) in this study. After 24 h of stimulation, the cells and culture supernatant were collected for further analyses. 

### 5.2. RNA-Seq and RT-qPCR Validation

Three cell samples from each group were randomly selected for RNA-Seq and post-sequencing bioinformatics analysis. The RNA-Seq was carried out using the Illumina HiSeq2500 platform. The details of sequencing procedures were provided by the technical service of Genedenovo Biotechnology Co., Ltd. (Guangzhou, China) (http://www.genedenovo.com/). The post-sequencing bioinformatics analysis was performed using the online OmicShare cloud platform (https://www.omicshare.com/tools/). The six inflammation-related genes and three casein genes were selected to validate RNA-Seq data by RT-qPCR. The primer sequences of genes are supplied in Table S4. Six cell samples from each group were harvested for total RNA extraction. The obtained total RNA was retrotranscribed for generating complementary DNA (cDNA). The cDNA was used for RT-qPCR on a fluorescent quantitative PCR system. The relative expression of target genes was calculated using the 2^−ΔΔCT^ method. The detailed methods for performing RT-qPCR were described in our previous study [19].

### 5.3. Western Blot and Enzyme-Linked Immunosorbent Assay (ELISA)

Nucleoprotein and total protein were extracted from the cell samples to detect histone acetylation and casein expression levels by Western blot, respectively. Briefly, the Western blot was performed mainly in six steps as follows: (1) protein sample preparation; (2) sodium dodecyl sulfate -polyacrylamide gel electrophoresis (SDS-PAGE); (3) protein transfer; (4) primary antibody and secondary antibody incubation; (5) protein band visualization; and (6) gray value calculation. The extracted nucleoprotein was also used to measure the activity of DNMT, HAT and HDAC by ELISA. Total DNA was extracted from the cell samples to determine global DNA methylation levels by ELISA. Medium supernatant was collected to detect inflammatory factor concentrations by ELISA. All the materials and methods used in the Western blot and ELISA were the same as those of our previous study, in which a detailed description is available [19].

### 5.4. Statistical Analysis

Statistical analysis was carried out using SPSS version 19.0 statistics software (SPSS, Chicago, IL, USA). One-way analysis of variance followed by Duncan’s multiple comparison test was employed to assess the significance of differences among the variables of the three groups. Significant difference was considered at a probability value of less than 0.05 (*p* < 0.05). The experimental data obtained are presented as means ± standard deviation (SD). 

## Figures and Tables

**Table 1:** The fold change in immune- or inflammation-related differentially expressed genes (DEGs) induced by LPS or PLL, compared with non-treatment.

Gene Symbol	Gene Description	Fold Change *	
		LPS	PLL
**Cytokines and Chemokines**			
*CCL20*	C-C motif chemokine 20	802.17	1266.00
*CCL5*	C-C motif chemokine 5	9.29	13.80
*CXCL1*	chemokine (C-X-C motif) ligand 1	6.34	9.52
*CXCL6*	chemokine (C-X-C motif) ligand 6	20.07	32.26
*CXCL8* (*IL-8*)	interleukin-8	26.24	54.95
*IL1A* (*IL-1α*)	interleukin-1 alpha	3.73	5.76
*IL1B* (*IL-1β*)	interleukin-1 beta	310.00	300.00
*IL6*	interleukin-6	5.06	8.06
*NFKBIA*	NF-kappa-B inhibitor alpha	2.42	3.19
*TNF*	tumor necrosis factor	34.00	62.00
*CSF2*	granulocyte-macrophage colony-stimulating factor	–	4.11
*Flt3lg*	fms-related tyrosine kinase 3 ligand isoform X2	–	2.08
*TNFSF10*	tumor necrosis factor ligand superfamily member 10	–	2.20
**Another Immune Associated DEGs**			
*C1S*	TPA: complement C1s subcomponent	2.13	-
*C2*	complement C2	4.44	6.44
*CFB*	complement factor B	52.12	132.50
*ISG15*	ubiquitin-like protein ISG15	2.16	11.60
*MMP9*	matrix metalloproteinase-9	2.85	4.89
*MAP2K6*	dual specificity mitogen-activated protein kinase kinase 6	–	0.46
*MAP3K8*	mitogen-activated protein kinase kinase kinase 8 isoform X2	–	2.77
*CD14*	monocyte differentiation antigen CD14 isoform X2	–	5.77
*ASAP3*	arf-GAP with SH3 domain, ANK repeat and PH domain-containing protein 3	–	3.20
*TLR3*	toll-like receptor 3 isoform X1	–	2.53
*MEFV*	pyrin isoform X1	–	10.67
*DHX58*	probable ATP-dependent RNA helicase DHX58	–	2.95
*DDX58*	probable ATP-dependent RNA helicase DDX58	–	2.10
*ZBP1*	TPA: Z-DNA binding protein 1-like	–	3.47
*CD3G*	T-cell surface glycoprotein CD3 gamma chain	–	0.47
*TNFAIP3*	tumor necrosis factor alpha-induced protein 3	–	2.17

* Data obtained by RNA-Seq and presented as the fold change (FC) of the DEGs induced by lipopolysaccharide (LPS) and co-stimulation with peptidoglycan (PGN), lipoteichoic acid (LTA), and LPS (PLL). For all the FCs shown, the false-discovery rate (FDR) is <0.05 and |log_2_ FC| > 1. “–” indicates that the gene did not belong to DEGs.

## Supplementary Materials

The following are available online at https://www.mdpi.com/2072-6651/12/6/387/s1, Table S1: The 35 common DEGs between the CON vs. LPS and CON vs. PLL; Table S2: The 9 common DEGs in the three conditions (CON vs. LPS, CON vs. PLL and LPS vs. PLL); Table S3: The 12 DEGs that only appeared in the condition of CON vs. LPS; Table S4: The primer sequences of target genes and an internal reference gene (GAPDH).
