# Supplementary material for: The Synergism of PGN, LTA and LPS in Inducing Transcriptome Changes, Inflammatory Responses and a Decrease in Lactation as Well as the Associated Epigenetic Mechanisms in Bovine Mammary Epithelial Cells
Source: Toxins (Basel). 2020 Jun 11;12(6):387. doi: 10.3390/toxins12060387 (PMC7354563; doi:10.3390/toxins12060387)
Supplement: Supplementary file 1 [file toxins-12-00387-s001.zip › supplementary materials/Table S4. The primer sequences of target genes and an internal reference gene (GAPDH).pdf]

Supplementary Materials: The Synergism of PGN, LTA and LPS in Inducing Transcriptome Changes, Inflammatory Responses and a Decrease in Lactation as Well as the Associated Epigenetic Mechanisms in Bovine Mammary Epithelial Cells

Yongjiang Wu, Yawang Sun, Xianwen Dong, Jingbo Chen, Zili Wang, Juncai Chen and Guozhong Dong

Table S4. The primer sequences of target genes and an internal reference gene (GAPDH).

Gene	Primer Sequence (5' to 3')	Product Size/bp	Accession Number
IL-1β	F: AGTGCCTACGCACATGTCTTC<br>R: TGCGTCACACAGAAACTCGTC	114	NM_174093.1
IL-6	F: TGCTGGTCTTCTGGAGTATC<br>R: GTGGCTGGAGTGGTTATTAG	153	NM_173923.2
IL-8	F: ATGACTTCCAAGCTGGCTGTTG<br>R: TTGATAAATTTGGGGTGGAAAG	149	NM_173925.2
CXCL1	F: ACCTCAAGAACATCCAGAGCG<br>R: GCTGGAGTATCAAGAAGCTCGT	203	NM_175700.2
CXCL6	F: CCTCTGCAGTCCTCTCTTCG<br>R: TGGGATGAATTCCCGGTGTG	203	NM_174300.2
TNF-α	F: CCACGTTGTAGCCGACATC<br>R: CCCTGAAGAGGACCTGTGAG	155	XM_005223596.4
CSN1S1	F: CTTTTCAGACAATTCTACCAGCT<br>R: AATTCACTTGACTCCTCACCAC	171	NM_181029.2
CSN2	F: AGTCCAAAGTCCTGCCTGTTCC<br>R: TGCCATATTTCCAGTCGCAGTC	193	XM_015471671.2
CSN3	F: CAATACGCTGTGAGAAAGATGA<br>R: AACTGGTTTCTGTTGGTAGTAA	122	NM_174294.2
GAPDH	F: GGGTCATCATCTCTGCACCT<br>R: GGTCATAAGTCCCTCCACGA	176	NM_001034034.2

IL-1β, interleukin-1β; *IL-6*, interleukin-6; *IL-8*, interleukin-8; *CXCL1*, chemokine (C-X-C motif) ligand 1; *CXCL6*, chemokine (C-X-C motif) ligand 6; *TNF-α*, tumor necrosis factor-α; *CSN1S1*, αS1-casein; *CSN2*, β-casein; *CSN3*, κ-casein; *GAPDH*, glyceraldehyde-3-phosphate dehydrogenase.
